# The impact of social prescribing on well-being outcomes in a nationwide analysis

**DOI:** 10.1038/s44360-026-00099-w

**Published:** 2026-03-24

**Authors:** Feifei Bu, Daniel Hayes, Luke Munford, Daisy Fancourt

**Affiliations:** 1 Social Biobehavioural Group, Research Department of Behavioural Science and Health, Institute of Epidemiology and Health Care, University College London, London, UK; 2 School of Health Sciences, University of Manchester and NIHR Applied Research Collaboration Greater Manchester, Manchester, UK

**Keywords:** Health services, Health policy

## Abstract

Social prescribing (SP) is growing rapidly in the UK and across the world. However, there remains a lack of robust quantitative evidence on its impacts. Here we aim to provide a large-scale national analysis of the well-being impacts of SP in the UK, drawing pre–post data from large-scale longitudinal administrative records. Well-being outcomes were measured using the short Warwick–Edinburgh Mental Wellbeing Scale and the Office for National Statistics well-being scale, including happiness, anxiety, life satisfaction and sense of life being worthwhile. Data were analysed using Bayesian growth curve modelling. Our findings demonstrate consistent and sizeable improvements across different well-being domains in the 1–6 months following initial referral to SP. Specifically, we observed a 3.31-point increase in the short Warwick–Edinburgh Mental Wellbeing Scale (95% highest density interval (HDI) 3.26 to 3.37), a 1.59-point increase in happiness (95% HDI 1.55 to 1.63), a 1.57-point increase in life satisfaction (95% HDI 1.54 to 1.61), a 1.4-point increase in sense of life being worthwhile (95% HDI 1.36 to 1.43) and a 1.45-point decrease in anxiety (95% HDI −1.49 to −1.41). The increase in life satisfaction is equivalent to a conservative monetary estimate of £4,252 over a mean period of 2.5 months (2019 prices), representing a return on investment of £9 (per £1 invested). These results proved robust across multiple sensitivity analyses. We found little evidence that the well-being changes differ across sociodemographic groups, indicating a broad applicability of SP interventions.

## Abstract (web summary)

In a quasi-experimental study, referral to community-based, non-medical resources, commonly known as ‘social prescribing’, improved scores related to life satisfaction and decreased symptoms of anxiety in a nationally comprehensive sample of 19,627 patients from the UK.

## Main

Social prescribing (SP) is a mechanism of care that connects individuals to non-medical forms of support, based in their local communities^1^. This definition aligns with the broader conceptual and operational framework in the international literature, which further emphasizes its person-centred and coproductive nature and the role of a trusted individual involved in the connection process^2^. Following the pioneering nationwide programme in England, SP has rapidly developed globally in the past decade, with varying delivery models^1,2^. The most common model in England is the general practitioner link worker model, in which link workers are employed through the Additional Roles Reimbursement Scheme to work directly with referred patients to codevelop a non-clinical plan based on the values, needs and preferences of patients, connecting them with existing community activities. These activities can include (but are not limited to) the arts, music, access to nature, volunteering, gardening, exercise and broader support services^3^. In addition to the predominant general practitioner link worker model, individuals can also be referred via alternative routes, such as secondary care, voluntary and community sector organizations, statutory services and self-referral. The importance of SP lies in its potential to address the social, emotional and practical needs of patients that are often interrelated to medical needs but are not covered by clinical treatments, and which form an estimated 19% of general practitioner consultation time^4^. When incorporated into healthcare, SP can serve as a preventative approach, addressing challenges such as social isolation and loneliness either before physical and mental health symptoms arise or before they progress. Alternatively, it can be embedded into a patient’s healthcare plan alongside other treatments to provide a holistic model of care^2^.

So far, the evidence base for SP suggests an overall positive impact on well-being outcomes. A recent qualitative meta-synthesis across 18 studies of 1,506 patients who received SP reported that SP increased well-being via aspects such as increasing a sense of belonging to the community, increasing confidence and self-worth and providing a sense of purpose, pride and achievement^5^. Quantitative metrics also suggest increases in well-being, with a recent systematic review identifying five studies where well-being was measured, of which four showed large effect sizes over a short follow-up period, while the other showed no effect over an intermediate follow-up period^6^. However, while helpful, these reviews are not without limitations. The qualitative meta-synthesis included studies of varying quality, with participants being predominantly older adults and female, leaving it unclear whether other populations perceive similar benefits. Similarly, for the quantitative systematic review, studies also ranged in methodological quality, with most scoring weak ratings overall on quality assessment. All five well-being studies used an uncontrolled before–after study design. Three studies^7–9^ had sample sizes under 150 participants for pre–post outcome analyses, suggesting that they were likely to be underpowered to detect an effect. In addition, as most studies focused exclusively on specific services or local areas, their findings cannot be generalized to broader services or patient populations. Last, the largest study (*n* = 1,297) examined only arts referrals via a local primary care setting^10^, leaving an evidence gap regarding how SP referrals via non-primary care routes or to other non-arts services affect well-being.

Clearly, a key challenge in SP research is balancing different study designs. In light of the methodological limitations identified with the previous studies outlined above, a number of controlled and randomized trials on SP are now underway^11,12^. While these will be critical for confirming causality, it is also important that national services are evaluated using large-scale administrative data that can capture real-world heterogeneity in service delivery and provide large samples enabling consideration of potential moderation by sociodemographic characteristics. In addition, given the current financial pressures experienced by the UK and other governments, it is particularly important to consider the opportunity costs and value for money of SP to support commissioning decisions.

In the UK, HM Treasury recommends that policies and interventions—such as SP link workers—are appraised using the Green Book approach^13^. In particular, well-being should be incorporated by using the widely collected Office for National Statistics well-being scale (ONS4) questions and translating the ‘life satisfaction’ question into well-being-adjusted life years (WELLBYs)^14^, which can be easily compared across alternative policies to determine value for money though metrics such as return on investment (RoI) or cost–benefit analysis. So far, the only large-scale analysis of WELLBYs has come from a national evaluation of green SP, focusing on nature-based activities in the UK. Findings from this study showed statistically significant improvements in well-being as measured by the ONS4 across 3,339 individuals, with a 1.9-point increase in happiness, a 1.7-point increase in life satisfaction, a 1.0-point reduction in anxiety and a 1.3-point increase in sense of worthwhileness^15^. From this, the authors estimated the social return on investment was £1.88–2.42 per £1 invested. However, in line with previous work, its narrow focus on nature-based activities means that well-being effects to other referrals are uncertain.

Thus, to address the critical gap, the present study leveraged data from administrative records obtained from Access Elemental, a digital SP platform used by health and social care professionals, community development workers and other service providers to keep track of SP activities and their impact from the point of referral. Although Access Elemental does not comprise a random sample of sites or patients in the UK, it does have good geographical coverage in England (45% of integrated care boards), Wales (57% of Welsh university health boards) and Northern Ireland (80% of health and social care trusts), alongside smaller representation in Scotland (13% of Scottish health and social care partnerships). A challenge with the Access Elemental dataset is that it only contains records for patients who received SP, precluding the creation of non-referred counterfactuals possible in other primary care data sources. However, compared with primary care data, Access Elemental data have the major advantages of multi-stakeholder input, allowing for examining other SP pathways in addition to the general practitioner link worker model and richer information on details of referrals including embedded pre–post outcome measures in some sites. As such, while no dataset can provide a complete picture of SP practice, Access Elemental provides an important data source to triangulate with data from local evaluations and experimental studies. Further, to strengthen the robustness of analyses, we applied more sophisticated statistical analyses than previously used for exploration of SP, employing growth curve modelling within the Bayesian framework, which provides more flexibility for modelling pre–post longitudinal data.

Specifically, our study aimed to (1) examine changes in well-being before and after SP, comparing outcomes across multiple well-being domains; (2) assess potential heterogeneous impacts of SP by individual characteristics; and (3) quantify well-being improvements in monetary terms and offer some insights into the RoI. These aims are critical in building the evidence base for SP considering its global proliferation and the pivotal moment for the National Health Service (NHS) in transforming the health and care system towards neighbourhood health.

## Results

### Demographics

The short Warwick–Edinburgh Mental Wellbeing Scale (SWEMWBS) and ONS4 analytical samples were broadly comparable to the overall Access Elemental patient cohort regarding gender, age and area deprivation (Table 1). There were, however, some imbalances in geography and referral route. Specifically, patients from rural areas were overrepresented in the SWEMWBS sample (19.9% versus 12.1%) but underrepresented in the ONS4 sample (8.1% versus 12.1%). Similarly, there was an overrepresentation of patients from Northern Ireland in the SWEMWBS sample (16.1% versus 4.7%), but an underrepresentation in the ONS4 sample (0.6% versus 4.7%). In both SWEMWBS and ONS4 samples, there was an underrepresentation of patients from a non-medical route. The majority of data (>60%) were collected between 2021 and 2024.

**Table 1:** Sample characteristics of the SWEMWBS (*N* = 19,627) and ONS4 (*N* = 14,657) analytical samples compared with the full Access Elemental cohort (*N* = 601,529)

Variables	Analytical sample SWEMWBS		Analytical sample ONS4		Full Access Elemental cohort	
	Valid *N*	Percentage	Valid *N*	Percentage	Valid *N*	Percentage
Gender: female	19,274	64.1%	14,484	65.9%	584,255	61.8%
Gender: male	19,274	35.9%	14,484	34.1%	584,255	38.2%
Age: 29 years or under	19,558	15.5%	14,656	12.4%	597,684	15.2%
Age: 30–49 years	19,558	31.6%	14,656	30.7%	597,684	31.6%
Age: 50–69 years	19,558	36.4%	14,656	36.7%	597,684	31.9%
Age: 70+ years	19,558	16.5%	14,656	20.2%	597,684	21.3%
IMD: 1 (most deprived)	17,394	42.0%	11,937	36.3%	468,524	37.1%
IMD: 2	17,394	21.0%	11,937	21.0%	468,524	22.3%
IMD: 3	17,394	16.0%	11,937	17.1%	468,524	16.5%
IMD: 4	17,394	13.3%	11,937	15.7%	468,524	14.2%
IMD: 5 (least deprived)	17,394	7.8%	11,937	9.9%	468,524	9.9%
Area: rural	17,394	19.9%	11,937	8.1%	468,524	12.1%
Area: urban	17,394	80.1%	11,937	91.9%	468,524	87.9%
Referral route: non-medical	16,939	10.1%	14,416	8.5%	542,663	17.1%
Referral route: medical	16,939	89.9%	14,416	91.5%	542,663	82.9%
Country: England	19,597	68.3%	14,626	88.0%	600,001	82.5%
Country: Wales	19,597	5.8%	14,626	8.8%	600,001	10.1%
Country: Scotland	19,597	9.8%	14,626	2.6%	600,001	2.7%
Country: Northern Ireland	19,597	16.1%	14,626	0.6%	600,001	4.7%
Case year: 2018 or before	19,627	1.8%	14,657	0.0%	601,529	2.0%
Case year: 2019	19,627	7.4%	14,657	0.4%	601,529	3.8%
Case year: 2020	19,627	7.3%	14,657	5.3%	601,529	7.5%
Case year: 2021	19,627	16.3%	14,657	15.1%	601,529	14.3%
Case year: 2022	19,627	20.0%	14,657	26.4%	601,529	21.1%
Case year: 2023	19,627	21.9%	14,657	24.8%	601,529	22.8%
Case year: 2024	19,627	22.8%	14,657	24.9%	601,529	21.4%
Case year: 2025	19,627	2.6%	14,657	3.2%	601,529	7.2%

The unit of *N* for the SWEMWBS and ONS4 analytical samples is patient (only one case per patient was kept), whereas the unit of *N* for all data is case (one patient may have more than one SP case) considering that some variables (for example, age, referral route and case year) are at case level.

### Well-being changes

**Fig. 1:**
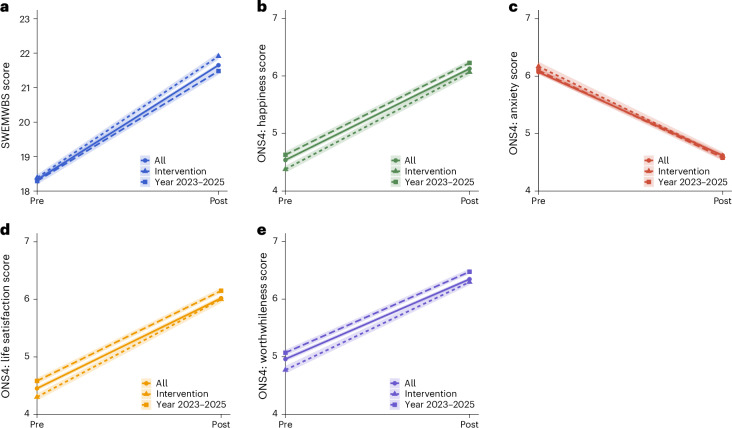
Results from unconditional Bayesian growth curve models. **a**–**e**, The predicted average trajectories and their 95% HDIs for SWEMWBS scores (**a**) and ONS4 measures of happiness (**b**), anxiety (**c**), life satisfaction (**d**) and sense of worthwhileness (**e**).

Figure 1 shows the estimated average growth trajectories and their 95% highest density intervals (HDIs) from unconditional growth models (also see Supplementary Table 1). On average, SWEMWBS increased by 3.31 points (95% HDI 3.26 to 3.37) between two timepoints within 1–6 months. Happiness increased by 1.59 (95% HDI 1.55 to 1.63), life satisfaction by 1.57 (95% HDI 1.54 to 1.61) and sense of worthwhileness by 1.4 points (95% HDI 1.36 to 1.43), while anxiety decreased by 1.45 points (95% HDI −1.49 to −1.41).

### Monetization

Using monetization methods, a 1.57-point increase in life satisfaction over a mean period of 2.5 months was equivalent to £4,252 in 2019 prices (low, £3,271; high, £5,233). If restricting to the intervention subsample, the monetized value of the 1.69-point increase was £4,577 (low, £3521; high, £5,633). On the basis of this and the Personal Social Services Research Unit (PSSRU) estimates, the RoI for SP is estimated to be £4,252/£466 = £9. Specifically, every pound invested in SP is likely to accrue £9 (low, £7; high, £11) in well-being benefits.

### Sensitivity analyses

Restricting analyses to the intervention subsample yielded marginally faster rates of increase or decrease compared with the main analyses (Supplementary Table 1). For example, life satisfaction was estimated to increase by 1.69 points (95% HDI 1.64 to 1.75), compared with 1.57 in the main analysis. When limited to 2023–2025, the change rates were similar to those in the main analyses (Supplementary Table 1). Notably, however, levels of happiness, life satisfaction and sense of worthwhile were consistently higher in the 2023–2025 subsample. Finally, sensitivity analyses adjusting entropy-balancing weights yielded results consistent with the main analyses (Supplementary Fig. 3).

### Subgroup differences

**Fig. 2:**
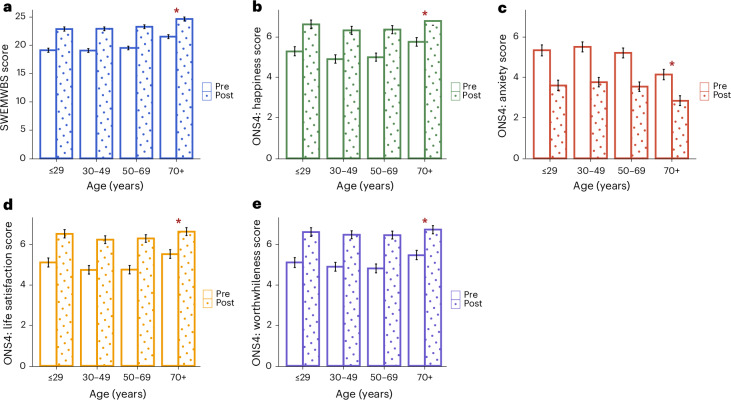
Results from conditional Bayesian growth curve models. **a**–**e**, Predicted pre and post well-being measures and their 95% HDIs by age groups for the SWEMWBS scores (**a**) (*N* = 15,001) and ONS4 measures of happiness (**b**), anxiety (**c**), life satisfaction (**d**) and sense of worthwhileness (**e**) (*N* = 11,720).

**Fig. 3:**
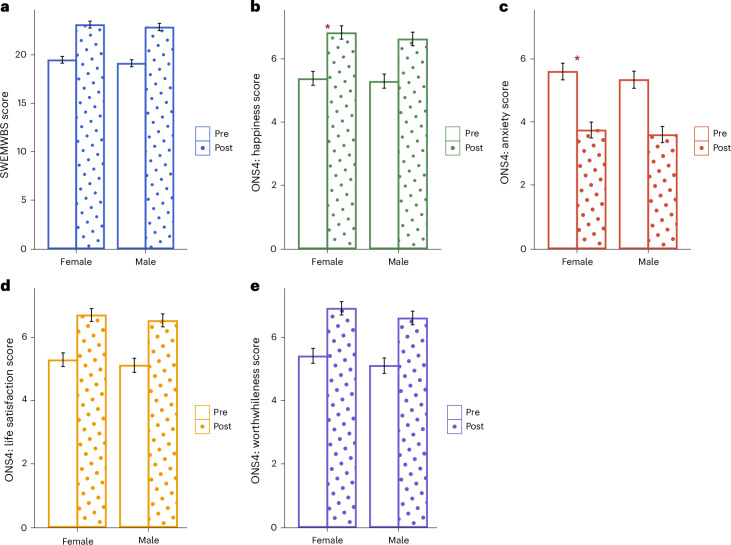
Results from conditional Bayesian growth curve models. **a**–**e**, Predicted pre and post well-being measures and their 95% HDIs by gender for the SWEMWBS scores (**a**) (*N* = 15,001) and ONS4 measures of happiness (**b**), anxiety (**c**), life satisfaction (**d**) and sense of worthwhileness (**e**) (*N* = 11,720).

Based on conditional growth models, whereas well-being improved across all age groups, older adults aged 70 years or over had consistently slower improvement across all outcomes compared with younger adults under 30 years (Fig. 2). This pattern aligns with the negative intercept–slope covariance, considering that older adults had higher levels of baseline well-being. There was some evidence that improvement rates differed by sex for happiness and anxiety, although the differences were only marginal (Fig. 3). There was little evidence that well-being improvements differed by area deprivation, urbanicity or referral route (Supplementary Table 2).

## Discussion

Analysing data from 14,657–19,627 patients from over 300 SP sites, our study provided a large-scale national analysis of the well-being impacts of SP in the UK. Our findings demonstrate consistent and sizeable improvements across different well-being measures, including general mental well-being measured by SWEMWBS and affective (happiness and anxiety), evaluative (life satisfaction) and eudemonic (worthwhileness) well-being measured by ONS4. The increase in life satisfaction is equivalent to a conservative monetary estimate of £4,252 over a mean period of 2.5 months (2019 prices), representing a RoI of £9. We found little evidence that the well-being changes differ across sociodemographic groups, indicating a broad applicability of SP interventions.

Overall, our study support findings from previous work, showing a positive impact of SP on well-being outcomes^5,6,15^. Notably, the magnitudes of well-being changes in our study are largely comparable to those found in the green SP study using ONS4 measures, despite its specific focus and differential analytical methodology^15^. As an uncontrolled study, our findings are susceptible to attention effects or other sources of bias. However, the observed well-being improvements are supported by evidence from randomized controlled trials of referrals to community-based activities commonly used in SP, such as arts and cultural or voluntary sector engagements^16,17^. This corroborating evidence strengthens the plausibility of an intervention effect. Regarding the timeframe for well-being impact, our study’s mean follow-up of 2.5 months (1–6 months) aligns with previous studies using SWEMWEBS or WEMWEBS measures (1.9–4 months)^6^, providing evidence for well-being impacts over short and intermediate durations. It remains unclear whether well-being improvements are sustained over longer periods, although previous trials of SP interventions suggest maintenance does occur^18^. Compared with previous work, our study expands on the existing literature by drawing on a large national dataset, which is not limited to specific geographical locations, services or specific referral schemes. Thus, it addresses the limitations of previous work and, in doing so, highlights the broad application on SP in improving well-being outcomes across different populations and contexts.

In relation to the health economics specifically, our findings suggest that SP yields substantial RoIs, contributing to a growing economic evidence base for SP^19,20^. The 1.57-point increase in life satisfaction translates to a monetary value of £4,252 (2019 price), resulting in a £9 RoI (low, £7; high, £11). While this is a conservative estimate based solely on life satisfaction, the resulting RoI is larger than those reported in most prior small-scale studies focusing on specific interventions or patient groups^15,21,22^, with the exception of one study reporting a range of £9–23 (ref. ^23^). Our RoI calculation was based on the most recent PSSRU data for SP referral costs, but it is important to acknowledge that the underlying PSSRU data was derived from a relatively small-scale pilot study. As cost estimates of SP continue to evolve, the RoI calculation can be amended using newer values of average costs per referral. In addition, the RoI only considers well-being as the measure of benefit. There may be other aspects of people’s lives that positively benefit from SP that could accrue additional benefits yet they are omitted here. Further, it is also important to acknowledge that, unlike frameworks such as social RoI, the WELLBY metric lacks formal adjustments for attribution, deadweight loss and displacement. Taken together, these economic findings should be interpreted with caution.

One of the major strengths of our study is analysing routine data collected in a large-scale cloud-based platform that enables tracking of diverse SP referral pathways, not just those confined to general practitioner link worker models. However, continued efforts to improve data collection and quality are needed to fully realize their potential. Although SWEMWBS and ONS4 are the most used well-being measures, these well-being data were only recorded for one-seventh of patients and fewer than 5% of patients had pre–post measures. Insight from sites suggests that the reasons for this are largely driven by whether SP sites have local-level mandates to record outcome data, meaning that the missing data can, to some extent, be considered ‘missing at random’ in that they are not determined by patient selection into monitoring. That said, it may be that sites that are more advanced in their SP delivery are more likely to carry out pre–post outcome monitoring. The 2023 NHS England Social Prescribing Information Standard mandates that SP data are captured on patient demographics, needs and concerns, support offered (including referred to activities) and outcomes^24^. This monitoring requirement has been reiterated in further documents, such as NHS England’s reference guide for primary care networks, giving the responsibility specifically to link workers^25^. However, it is evidently not being routinely followed as not only were outcome data only available in some sites, but there were high rates of missingness in key measures, such as ethnicity and time that link workers spent with patients. In our analytical sample, only 39–53% of patients had an intervention referral recorded between the pre–post timepoints, making it unclear whether others did not receive an intervention referral or it was merely unrecorded. Even when pre–post outcome measures were reportedly collected at initial and final sessions, substantial variations exist across link workers in the methods (for example, telephone, face to face, email, home visit, office visit and so on) and timing of data collection. These ambiguities and inconsistencies significantly undermine our ability to conduct more informed analyses of how implementation factors affect outcomes. Consequently, promoting systematic outcome data collection in the future is crucial not only for service evaluation but also for evidence-based service improvement. Achieving this consistently at a national scale probably requires formal commissioning requirements, supported by appropriate incentives or penalties to ensure compliance.

Other strengths of our study include robust analytical approaches and cross-validation using multiple well-being measures covering different domains. However, several limitations should be noted. First, our analyses are restricted to subsamples of the Access Elemental cohort with pre–post well-being measures. While the analytical samples are shown to have comparable sociodemographic profiles to the whole cohort, we cannot rule out the possibility of selection bias related to unmeasured characteristics or due to the overall representability of the Access Elemental cohort. Particularly, if patients who are more motivated or who have benefitted more from the service are more likely to remain contactable for follow-ups, the observed effects could be overestimated. Second, as discussed above, our study does not have any control group. A major limitation in uncontrolled pre–post design is regression to the mean, a statistical artefact that occurs when participants have extreme baseline values, leading to a natural decline when retested even without any intervention^26^. However, our analyses account for baseline-dependent growth by allowing for correlations between random intercepts and slopes and also allow us to inspect person-specific changes, in addition to the average rate of change, which mitigates some underlying causes of regression to the mean. The lack of a control group also limits our ability to rule out confounding by temporal trends. Nevertheless, this concern is likely to be mitigated by the extended study period, with patients receiving SP at different times across almost 7 years (2018–2025). Further, biases may rise from social desirability when patients are aware of the study hypotheses. However, the minimum 4-week interval between assessments may reduce this bias as patients are less likely to recall their initial responses precisely. Given these constraints, future research is needed to validate the present findings using more robust methods such as quasi‑experimental designs or randomized controlled trials.

Our study substantially advances the evidence on SP impacts, demonstrating improvements across multiple well-being measures using national data. We find that these improvements are largely found consistently across sociodemographic groups. This indicates a broad applicability of SP in line with it being a personalized approach that focuses on what matters most to the patient, with interventions tailored to their specific needs and preferences. Based on the estimates from this study and PSSRU unit costs on SP referrals, we estimated that the RoI was £9; that is, every pound invested in SP is associated with £9 in well-being benefits. Triangulation of these findings with other data from electronic patient health records and clinical trials will be critical to reaching a consensus on the likely impact and value for money of investment in SP services.

## Methods

### Data

So far, Access Elemental has data of over 600,000 SP cases from 482,575 patients (accessed on 25 April 2025). In this study, data were restricted to a subset of patients with repeated well-being measures between two timepoints within 1–6 months between 2017 and 2025 in the UK, with a mean follow-up of 2.5 months (s.d. of 1.2 months). For a small number of patients with more than one SP case recorded (~2–4%), the first case was used. The analytical sample sizes ranged from 14,657 to 19,627 for different well-being measures, with patients from over 300 SP sites covering all countries and regions in the UK (Supplementary Fig. 1). Sample selection diagrams are provided in Supplementary Fig. 2. All informed consent procedures were administered by the individual SP sites during service delivery. Access Elemental secured opt-out consent from these sites for anonymized data sharing. The research project was approved by the UCL Research Ethics Committee (approval no. 23909/002).

### Measurements

#### Well-being

The most commonly used well-being measures in the Access Elemental data were the SWEMWBS and ONS4. The SWEMWBS is a widely used and validated tool for measuring mental well-being and detecting clinically meaningful changes^27,28^. It includes seven positively worded items with five response categories from ‘none of the time’ to ‘all the time’. A total score was generated by summing the scores across seven items, which was then transformed into metric scores, ranging from 7 to 35 (ref. ^29^). The ONS4 questions cover happiness (affective well-being), anxiety (affective well-being), life satisfaction (evaluative well-being) and worthwhileness (eudemonic well-being), each measured on an 11-point Likert scale (0–10)^30^.

#### Covariates

Sociodemographic covariates included self-reported gender (female or male), age (under 30, 30–49, 50–69 or 70+ years), index of multiple deprivation (IMD; quintiles: 1: most deprived to 5: least deprived) and urbanicity (urban or rural). Both area deprivation and urbanicity were derived via postcode linkage to the urban rural classification and IMD at the lower-layer super output area in each country. We also included the referral route, indicating whether patients were referred from medical (for example, general practitioner or secondary care) or non-medical routes (for example, education or local authority). Although ethnicity was available as an optional measure, it had a high missing rate (>83%) and so was excluded from the analysis.

#### Monetization and RoI

As a key WELLBY metric, a one-point change in life satisfaction per year on the 0–10 scale is assigned a monetary value of £13,000, with a low-high range of £10,000–16,000 in 2019 prices based on the analysis published in the HM Treasury Green Book^14^. As our follow-up period was 2.5 months, we recalculated the monetary value for this time period rather than for the 12 months it typically represents, although this reflects a conservative estimate given analyses from previous studies suggest effects are maintained for longer. To calculate the RoI, we used the most recent PSSRU Costs of Health and Social Care, which estimate the cost per person for a SP referral to be £466 (ref. ^31^).

### Statistical analysis

Data were analysed using Bayesian growth curve modelling. This allowed us to estimate average pre–post changes while accounting for heterogeneous trajectories across individuals and to obtain more robust estimates of variance–covariance structures through the incorporation of priors and full propagation of uncertainty. For the main analysis, we fitted an unconditional linear growth model to the full analytical sample for each outcome measure (SWEMWBS, *N* = 19,627; ONS4, *N* = 14,657). For the life satisfaction model, we mapped the average rate of change and its credible intervals to the WELLBY metric to estimate monetary values. We conducted sensitivity analyses restricting to patients with recorded intervention prescriptions between the pre–post data timepoints (SWEMWBS, *N* = 10,413; ONS4, *N* = 5,677) and the subsample in 2023–2025 excluding data collected before or during the COVID-19 pandemic (SWEMWBS, *N* = 9,276; ONS4, *N* = 7,738). Sensitivity analyses were also performed by adjusting entropy-balancing weights to address the characteristic imbalance between the analytical sample and the full cohort in gender, age, area deprivation, urbanicity, country and referral route (SWEMWBS, *N* = 15,001; ONS4, *N* = 11,720). Further analyses were conducted to examine whether changes of well-being differed by individual characteristics by fitting conditional growth models using data from the subsample without missing covariates (SWEMWBS, *N* = 15,001; ONS4, *N* = 11,720). The conditional growth models included all covariates simultaneously and their interactions with time. The Bayesian growth curve models were fitted using non-informative priors, 2000 iterations, a burn-in of 1,000, a thinning of 5 and using Markov Chain Monte Carlo algorithms, implemented in JAGS. All analyses were conducted in in R 4.4.1.

### Reporting summary

Further information on research design is available in the Nature Portfolio Reporting Summary linked to this article.

## Supplementary information

Supplementary Information Supplementary Tables 1 and 2 and Figs. 1–3.

Reporting Summary

## Data Availability

The data used in this study are subject to third-party restrictions, so they are not publicly available. Interested researchers may request access to the minimal dataset underlying the analyses from the corresponding author, subject to permission from the third-party data owner.

## Code availability

Example code used in the analysis is publicly available via GitHub at https://github.com/FFBU/ESRC-SP.
