# Supplementary material for: The impact of social prescribing on well-being outcomes in a nationwide analysis
Source: Nat Health. 2026 Mar 24;1(7):737–44. doi: 10.1038/s44360-026-00099-w (PMC13337486; doi:10.1038/s44360-026-00099-w)
Supplement: Supplementary file 1 — Supplementary Tables 1 and 2 and Figs. 1–3. [file 44360_2026_99_MOESM1_ESM.pdf]

nature health

Supplementary information

https://doi.org/10.1038/s44360-026-00099-w

The impact of social prescribing on well-being outcomes in a nationwide analysis

In the format provided by the authors and unedited

Supplementary Materials: The impact of social prescribing on wellbeing outcomes in a nation-wide analysis

Table S1. Results from unconditional Bayesian growth curve models on SWEMWBS, happiness, anxiety, life satisfaction and worthwhile

**Main analysis: all data**	SWEMWBS (N=19,627)		ONS: happiness (N=14,657)		ONS: anxiety (N=14,657)		ONS: life satisfaction (N=14,657)		ONS: worthwhile (N=14,657)	
	Coef.	95% HDI	Coef.	95% HDI	Coef.	95% HDI	Coef.	95% HDI	Coef.	95% HDI
**Fixed effects**										
Intercept	18.33	[18.27, 18.40]	4.54	[4.50, 4.58]	6.07	[6.03, 6.12]	4.45	[4.41, 4.49]	4.96	[4.92, 5.00]
Slope	3.31	[3.26, 3.37]	1.59	[1.55, 1.63]	-1.45	[-1.50, -1.41]	1.57	[1.54, 1.61]	1.40	[1.36, 1.43]
**Random effects**										
SD: intercept	4.06	[3.72, 4.5]	2.01	[1.69, 2.30]	2.26	[1.93, 2.64]	1.85	[1.67, 2.07]	2.11	[1.86, 2.32]
SD: slope	2.50	[1.41, 3.88]	1.49	[0.47, 2.26]	1.59	[0.41, 2.59]	1.00	[0.25, 1.70]	1.40	[0.45, 1.98]
Covariance: intercept, slope	-0.28	[-0.42, -0.14]	-0.53	[-0.62, -0.45]	-0.49	[-0.57, -0.38]	-0.53	[-0.76, -0.44]	-0.57	[-0.74, -0.51]
SD: level 1 residual	2.15	[1.16, 2.79]	1.13	[0.46, 1.62]	1.32	[0.40, 1.84]	1.20	[0.79, 1.44]	1.07	[0.60, 1.50]
**Sensitivity analysis: intervention only**	SWEMWBS (N=10,413)		ONS: happiness (N=5,677)		ONS: anxiety (N=5,677)		ONS: life satisfaction (N=5,677)		ONS: worthwhile (N=5,677)	
	Coef.	95% HDI	Coef.	95% HDI	Coef.	95% HDI	Coef.	95% HDI	Coef.	95% HDI
**Fixed effects**										
Intercept	18.39	[18.29, 18.48]	4.38	[4.32, 4.44]	6.18	[6.11, 6.25]	4.30	[4.24, 4.36]	4.77	[4.71, 4.84]
Slope	3.52	[3.45, 3.60]	1.69	[1.63, 1.76]	-1.57	[-1.64, -1.50]	1.69	[1.64, 1.75]	1.53	[1.47, 1.59]
**Random effects**										
SD: intercept	4.18	[3.77, 4.73]	1.99	[1.69, 2.35]	2.15	[1.86, 2.59]	1.98	[1.70, 2.26]	2.11	[1.87, 2.44]
SD: slope	2.16	[0.30, 3.98]	1.42	[0.52, 2.36]	1.29	[0.33, 2.54]	1.37	[0.46, 2.12]	1.32	[0.51, 2.23]
Covariance: intercept, slope	-0.31	[-0.45, -0.13]	-0.53	[-0.6, -0.43]	-0.50	[-0.67, -0.36]	-0.55	[-0.69, -0.47]	-0.61	[-0.81, -0.52]
SD: level 1 residual	2.23	[0.86, 2.94]	1.26	[0.53, 1.71]	1.56	[0.71, 1.93]	1.03	[0.33, 1.50]	1.19	[0.39, 1.59]
**Sensitivity analysis: year 2023-2025**	SWEMWBS (N=9,276)		ONS: happiness (N=7,738)		ONS: anxiety (N=7,738)		ONS: life satisfaction (N=7,738)		ONS: worthwhile (N=7,738)	
	Coef.	95% HDI	Coef.	95% HDI	Coef.	95% HDI	Coef.	95% HDI	Coef.	95% HDI
**Fixed effects**										
Intercept	18.29	[18.19, 18.38]	4.64	[4.59, 4.69]	6.10	[6.04, 6.15]	4.58	[4.53, 4.63]	5.07	[5.02, 5.12]
Slope	3.19	[3.11, 3.27]	1.60	[1.55, 1.65]	-1.52	[-1.57, -1.46]	1.58	[1.53, 1.62]	1.41	[1.37, 1.46]
**Random effects**										
SD: intercept	4.15	[3.78, 4.63]	1.86	[1.65, 2.18]	2.10	[1.93, 2.42]	1.96	[1.70, 2.17]	2.03	[1.83, 2.29]
SD: slope	2.00	[0.27, 3.62]	1.10	[0.35, 1.99]	0.88	[0.22, 1.94]	1.40	[0.58, 1.98]	1.29	[0.60, 2.02]
Covariance: intercept, slope	-0.17	[-0.41, 0.14]	-0.54	[-0.72, -0.44]	-0.51	[-0.8, -0.38]	-0.52	[-0.57, -0.45]	-0.58	[-0.71, -0.52]
SD: level 1 residual	2.36	[1.35, 2.93]	1.27	[0.69, 1.55]	1.55	[1.05, 1.75]	0.87	[0.30, 1.38]	1.07	[0.43, 1.43]

Table S2. Results from conditional Bayesian growth curve models on SWEMWBS, happiness, anxiety, life satisfaction and worthwhile

	SWEMWBS (N=15,001)		ONS: happiness (N=11,720)		ONS: anxiety (N=11,720)		ONS: life satisfaction (N=11,720)		ONS: worthwhile (N=11,720)	
	Coef.	95% HDI	Coef.	95% HDI	Coef.	95% HDI	Coef.	95% HDI	Coef.	95% HDI
**Fixed effects**										
Intercept	19.12	[18.75, 19.48]	5.29	[5.07, 5.52]	5.35	[5.07, 5.61]	5.12	[4.89, 5.34]	5.11	[4.86, 5.35]
Slope	3.76	[3.41, 4.09]	1.35	[1.13, 1.55]	-1.75	[-2.00, -1.50]	1.42	[1.22, 1.64]	1.51	[1.31, 1.73]
Age: 30-49 (vs under 30)	-0.06	[-0.27, 0.15]	-0.38	[-0.53, -0.24]	0.17	[0.01, 0.33]	-0.37	[-0.51, -0.24]	-0.21	[-0.36, -0.07]
Age: 50-69 (vs under 30)	0.40	[0.20, 0.61]	-0.29	[-0.42, -0.15]	-0.13	[-0.29, 0.03]	-0.36	[-0.49, -0.23]	-0.30	[-0.44, -0.16]
Age: 70+ (vs under 30)	2.40	[2.15, 2.65]	0.47	[0.32, 0.63]	-1.21	[-1.39, -1.03]	0.41	[0.26, 0.55]	0.36	[0.20, 0.51]
Female (vs male)	0.35	[0.21, 0.49]	0.09	[0.00, 0.17]	0.26	[0.16, 0.36]	0.17	[0.09, 0.25]	0.30	[0.21, 0.39]
IMD: 2 (vs 1)	0.43	[0.24, 0.61]	0.06	[-0.05, 0.17]	0.06	[-0.07, 0.19]	0.15	[0.04, 0.26]	0.24	[0.12, 0.36]
IMD: 3 (vs 1)	1.17	[0.97, 1.38]	0.08	[-0.05, 0.20]	0.14	[0.00, 0.28]	0.17	[0.05, 0.28]	0.32	[0.19, 0.44]
IMD: 4 (vs 1)	1.77	[1.54, 1.99]	0.10	[-0.03, 0.23]	0.19	[0.05, 0.34]	0.28	[0.16, 0.40]	0.34	[0.21, 0.47]
IMD: 5 (vs 1)	1.33	[1.07, 1.61]	-0.03	[-0.18, 0.13]	0.38	[0.21, 0.55]	0.11	[-0.04, 0.25]	0.23	[0.08, 0.39]
Urban (vs rural)	-0.75	[-0.94, -0.57]	-0.32	[-0.47, -0.16]	0.49	[0.30, 0.67]	-0.22	[-0.37, -0.06]	-0.06	[-0.22, 0.1]
Medical (vs non-medical)	-1.97	[-2.21, -1.73]	-0.45	[-0.6, -0.31]	0.25	[0.08, 0.43]	-0.57	[-0.71, -0.43]	-0.42	[-0.58, -0.27]
Age: 30-49 (vs under 30) *slope	0.12	[-0.07, 0.31]	0.08	[-0.05, 0.23]	-0.01	[-0.16, 0.15]	0.08	[-0.04, 0.21]	0.08	[-0.05, 0.21]
Age: 50-69 (vs under 30) *slope	0.01	[-0.18, 0.21]	0.03	[-0.11, 0.16]	0.07	[-0.08, 0.22]	0.13	[0.00, 0.25]	0.15	[0.02, 0.28]
Age: 70+ (vs under 30) *slope	-0.61	[-0.85, -0.38]	-0.31	[-0.45, -0.16]	0.45	[0.28, 0.62]	-0.30	[-0.44, -0.17]	-0.24	[-0.38, -0.1]
Female (vs male) *slope	-0.11	[-0.25, 0.02]	0.11	[0.03, 0.20]	-0.11	[-0.21, -0.01]	0.01	[-0.07, 0.08]	0.01	[-0.07, 0.08]
IMD: 2 (vs 1) *slope	0.07	[-0.10, 0.25]	0.08	[-0.04, 0.18]	-0.07	[-0.19, 0.06]	0.03	[-0.08, 0.12]	-0.04	[-0.15, 0.06]
IMD: 3 (vs 1) *slope	-0.32	[-0.52, -0.12]	0.05	[-0.07, 0.17]	-0.03	[-0.17, 0.10]	0.06	[-0.05, 0.17]	-0.09	[-0.21, 0.02]
IMD: 4 (vs 1) *slope	-0.17	[-0.39, 0.04]	0.13	[0.01, 0.25]	-0.19	[-0.34, -0.05]	0.03	[-0.08, 0.14]	-0.06	[-0.18, 0.06]
IMD: 5 (vs 1) *slope	0.00	[-0.25, 0.26]	0.10	[-0.05, 0.25]	-0.17	[-0.34, 0.00]	0.01	[-0.12, 0.15]	-0.07	[-0.21, 0.07]
Urban (vs rural) *slope	-0.19	[-0.36, -0.01]	0.13	[-0.01, 0.28]	-0.02	[-0.19, 0.16]	0.07	[-0.07, 0.21]	-0.09	[-0.22, 0.06]
Medical (vs non-medical) *slope	-0.02	[-0.24, 0.21]	0.02	[-0.12, 0.16]	0.37	[0.20, 0.53]	0.06	[-0.07, 0.19]	-0.01	[-0.15, 0.13]
**Random effects**										
SD: intercept	3.55	[3.19, 4.25]	1.92	[1.64, 2.29]	2.24	[1.93, 2.55]	1.86	[1.65, 2.09]	2.05	[1.83, 2.31]
SD: slope	2.00	[0.64, 3.99]	1.26	[0.28, 2.26]	1.58	[0.54, 2.43]	1.16	[0.39, 1.83]	1.28	[0.55, 2.04]
Covariance: intercept, slope	-0.11	[-0.43, 0.29]	-0.56	[-0.81, -0.44]	-0.48	[-0.54, -0.4]	-0.50	[-0.6, -0.43]	-0.57	[-0.69, -0.5]
SD: level 1 residual	2.29	[0.62, 2.86]	1.12	[0.16, 1.6]	1.29	[0.63, 1.78]	1.10	[0.65, 1.42]	1.08	[0.46, 1.46]

a b

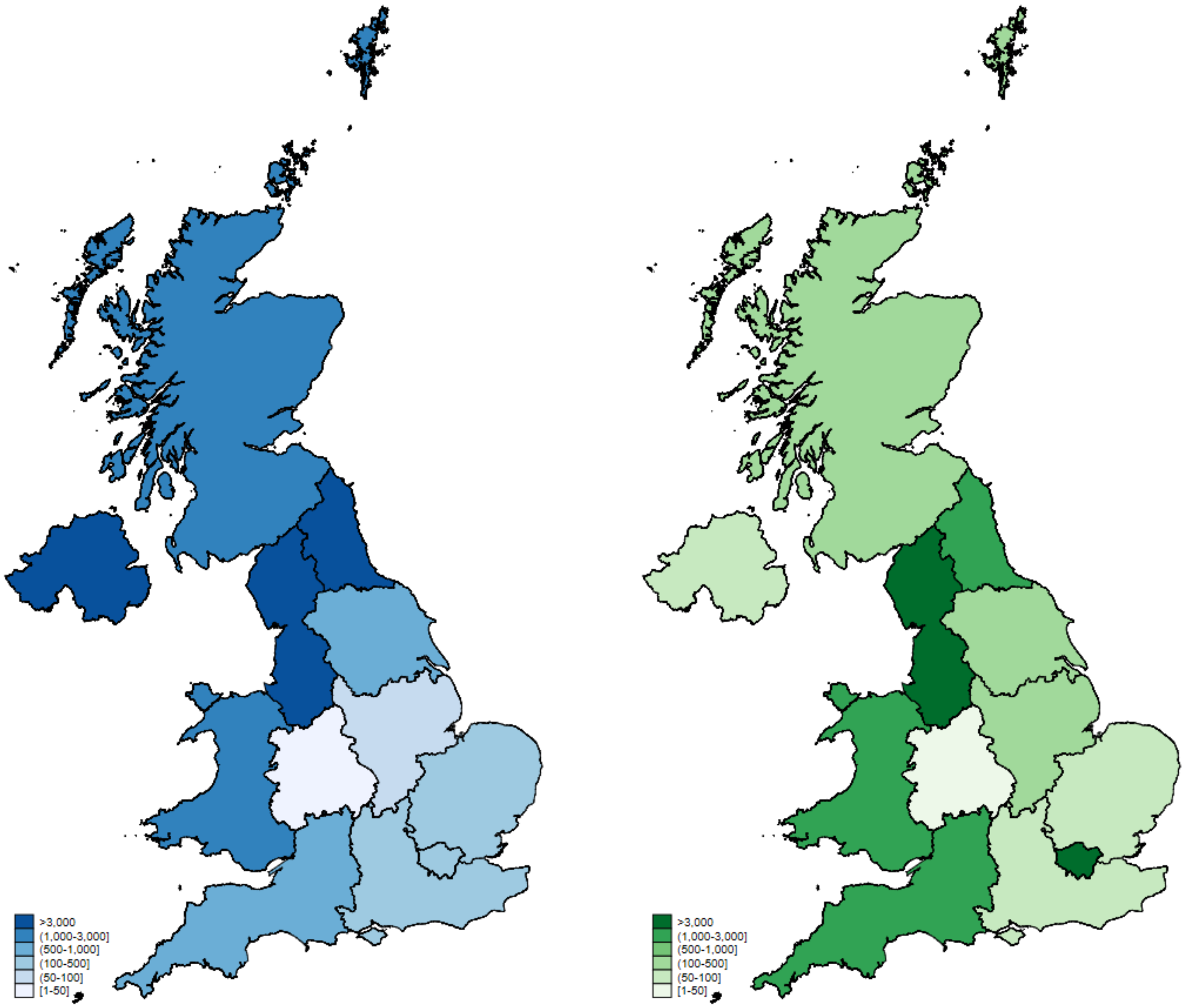

Source: Office for National Statistics licensed under the Open Government Licence v.3.0
Contains Ordnance Survey data © Crown Copyright 2007, License number 100017572.

Figure S1 Sample geographic distribution across countries and regions (a) SWEMWBS (b) ONS4

a

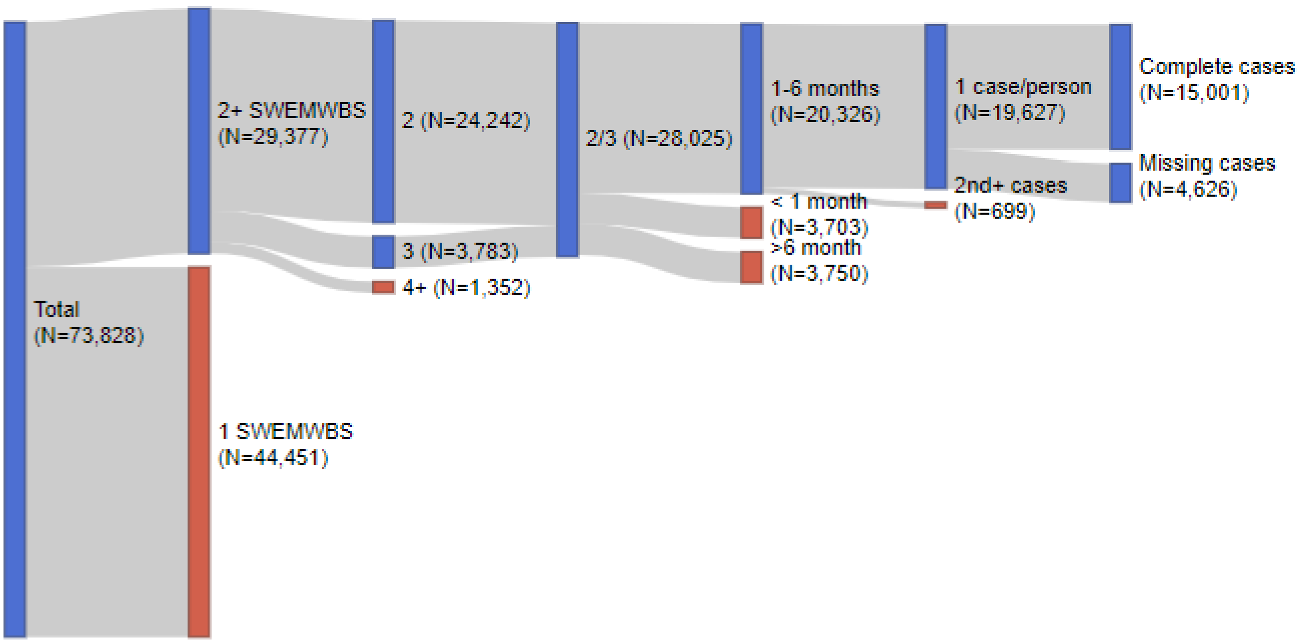

b

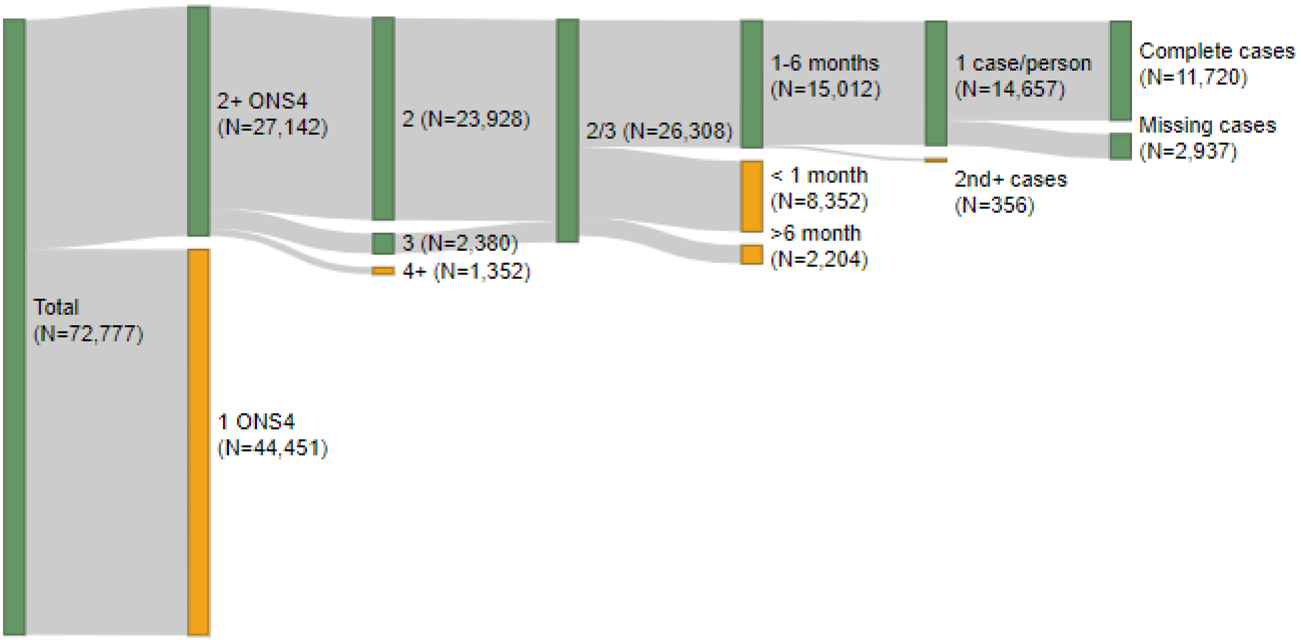

Figure S2 Sample selection diagram (a) SWEMWBS (b) ONS4

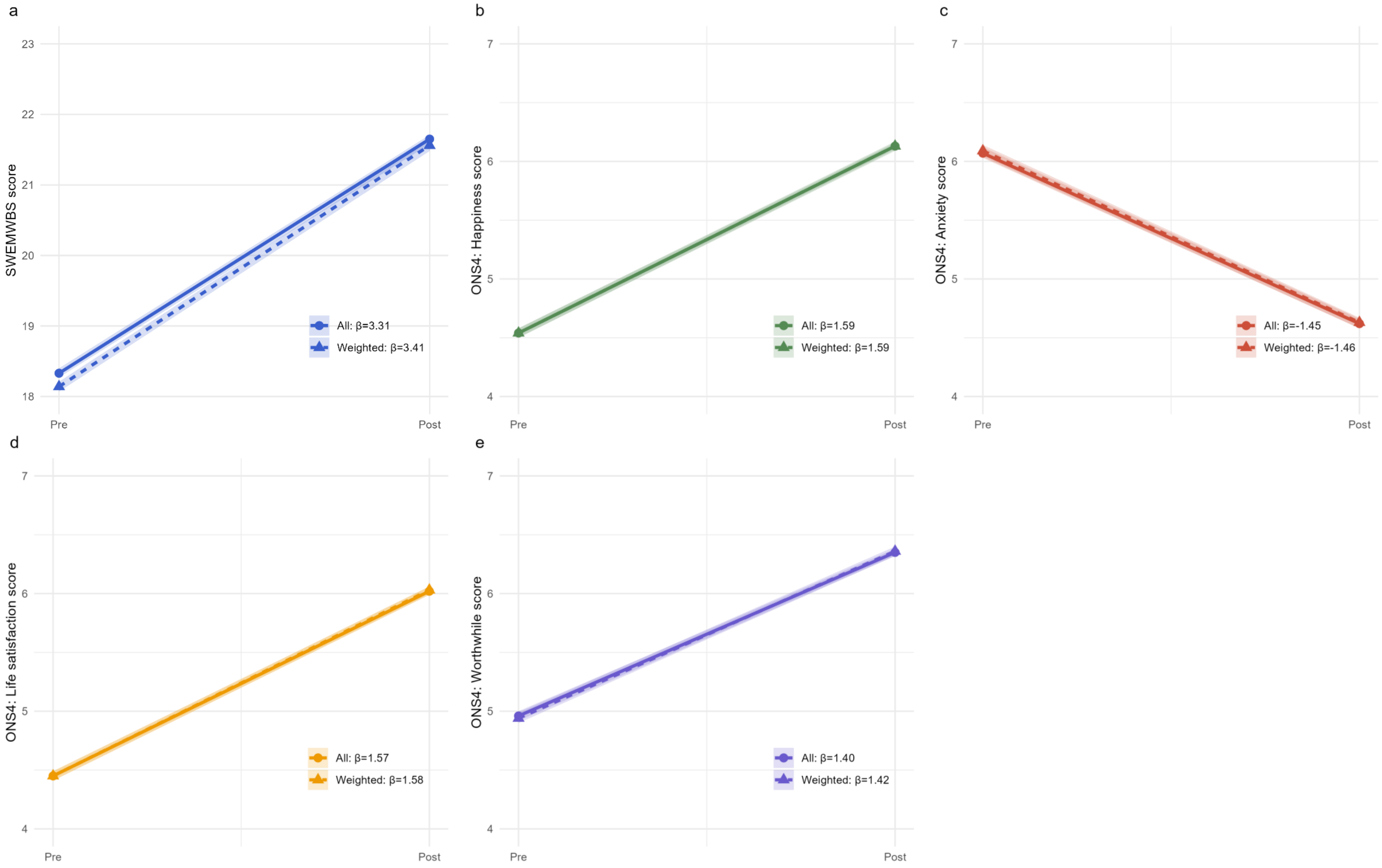

Figure S3 Predicted average trajectories and their 95% highest density intervals (HDI) from unconditional Bayesian growth curve models (weighted, SWEMWBS (a): N=15,001 ONS4 (b)-(e): N=11,720)
